# Residence in a Hispanic Enclave Is Associated with Inferior Overall Survival among Children with Acute Lymphoblastic Leukemia

**DOI:** 10.3390/ijerph18179273

**Published:** 2021-09-02

**Authors:** Jeremy M. Schraw, Erin C. Peckham-Gregory, Amy E. Hughes, Michael E. Scheurer, Sandi L. Pruitt, Philip J. Lupo

**Affiliations:** 1 Department of Pediatrics, Baylor College of Medicine, Houston, TX 77030, USA; erin.peckham-gregory@bcm.edu (E.C.P.-G.); scheurer@bcm.edu (M.E.S.); philip.lupo@bcm.edu (P.J.L.); 2 Texas Children’s Cancer and Hematology Centers, Texas Children’s Hospital, Houston, TX 77030, USA; 3 Department of Population and Data Sciences, University of Texas Southwestern Medical Center, Dallas, TX 75390, USA; amye.hughes@utsouthwestern.edu (A.E.H.); sandi.pruitt@utsouthwestern.edu (S.L.P.); 4 Harold C. Simmons Comprehensive Cancer Center, University of Texas Southwestern Medical Center, Dallas, TX 75390, USA

**Keywords:** acute lymphoblastic leukemia, childhood cancer, epidemiology, ethnic enclave, health disparities, social determinants of health, geographic information systems

## Abstract

Hispanic children with acute lymphoblastic leukemia (ALL) experience poorer overall survival (OS) than non-Hispanic White children; however, few studies have investigated the social determinants of this disparity. In Texas, many Hispanic individuals reside in ethnic enclaves—areas with high concentrations of immigrants, ethnic-specific businesses, and language isolation, which are often socioeconomically deprived. We determined whether enclave residence was associated with ALL survival, overall and among Hispanic children. We computed Hispanic enclave index scores for Texas census tracts, and classified children (*N* = 4083) as residing in enclaves if their residential tracts scored in the highest statewide quintile. We used Cox regression to evaluate the association between enclave residence and OS. Five-year OS was 78.6% for children in enclaves, and 77.8% for Hispanic children in enclaves, both significantly lower (*p* < 0.05) than the 85.8% observed among children not in enclaves. Children in enclaves had increased risk of death (hazard ratio (HR) 1.20, 95% confidence interval (CI) 1.01–1.49) after adjustment for sex, age at diagnosis, year of diagnosis, metropolitan residence and neighborhood socioeconomic deprivation and after further adjustment for child race/ethnicity (HR 1.19, 95% CI 0.97–1.45). We observed increased risk of death when analyses were restricted to Hispanic children specifically (HR 1.30, 95% CI 1.03–1.65). Observations suggest that children with ALL residing in Hispanic enclaves experience inferior OS.

## 1. Introduction

Acute lymphoblastic leukemia (ALL) accounts for 25% of cancers in children [1,2,3]. In the United States, it is diagnosed in approximately 3500 children per year and remains a leading cause of death among young people [1,3]. Hispanic children with ALL experience poorer overall and event-free survival compared to non-Hispanic Whites, with mortality rate ratios in excess of 1.5 reported [4,5,6,7]. The causes of this disparity are not fully understood, but both genetic and social factors may be implicated. For instance, Hispanic children tend be older than their non-Hispanic White peers at diagnosis [8] and display a different distribution of cytogenetic features, such as reduced frequency of favorable *ETV6-RUNX1* fusion and increased frequency of unfavorable *CRLF2* rearrangement, which likely contribute to poorer prognosis [9,10,11]. However, ethnic disparities may also be influenced by factors such as the high prevalence of obesity among Hispanic children [12,13], disproportionate difficulty managing the financial burden of a childhood cancer diagnosis among ethnic minority and/or socioeconomically disadvantaged families [14,15], differential access or adherence to care [16,17], and under-representation in clinical trials [18,19]. In addition to these individual factors, neighborhood socioeconomic deprivation is associated with inferior survival among children with ALL [20,21,22,23].

In the United States, race/ethnicity, socioeconomic position, and place of residence are interrelated [24,25], and many Hispanics reside in ethnic enclaves—neighborhoods characterized by high concentrations of recent immigrants from the same ethnic background, ethnic businesses, language isolation, and, often, socioeconomic deprivation [26,27]. In a population-based assessment performed in Texas and California, Shariff-Marco et al. reported that >60% of U.S.-born and >70% of foreign-born Latinas with breast cancer lived in neighborhoods classified as Hispanic enclaves [26]. Enclaves may exert both positive and negative effects on the health of their residents: while these neighborhoods often experience a high degree of material hardship and cultural isolation, residents may benefit from increased social support [26]. Perhaps because of the potentially complex effects of residence in an enclave or perhaps because of differences in populations and methods, studies of the association between residence in enclaves and survival in Hispanics with cancer report inconsistent findings. Evaluations of Latinas with breast cancer variously suggest that residence in an enclave is associated with increased [26,28] or decreased [29] survival. Hispanic enclaves have also been associated with superior survival for patients with colon [30] and prostate cancer [31], whereas no association was observed among Latinas with endometrial cancer [32]. Of note, no studies have assessed whether residing in an enclave is associated with survival among children with cancer.

We sought to determine whether residence in a Hispanic enclave was associated with survival among children with ALL, overall and among Hispanics specifically. Our investigation was motivated by the persistent survival disparity for Hispanic children with ALL, the lack of data concerning ethnic enclaves in this population, and the observation that, in the U.S. from 1992 to 2011, the Hispanic population experienced a larger annualized increase in ALL incidence than the non-Hispanic population [33]. These points underscore the critical need to understand and address factors associated with survival disparities in Hispanic children with ALL.

## 2. Materials and Methods

### 2.1. Study Sample

Our study included *N* = 4083 children with B-lineage ALL (International Classification of Childhood Cancer, Third Edition group 1.a.1) [34]. Eligible cases were <20 years of age, were diagnosed in Texas between 1995 and 2011, and resided in Texas at the time of diagnosis. The population-based Texas Cancer Registry (TCR) provided information on sex, race/ethnicity, International Classification of Disease for Oncology, Third Edition (ICD-O-3) site, histology, and behavior codes, residential address at diagnosis, vital status, and cause of death. TCR ascertained vital status through the end of the study period (31 December 2012) by linkage to the National Death Index, Social Security Death Index, and State Mortality File.

This study was approved by the Baylor College of Medicine Institutional Review Board (H-33217) on 23 January 2014.

### 2.2. Hispanic Enclave Index and Area Deprivation Index Scores

We used a modified version [35] of the Hispanic enclave index that is commonly applied in cancer epidemiology studies [36]. This four-dimensional measure is derived from principal components analysis of four component variables: percentages of residents who are Hispanic, are Hispanic and foreign-born, have limited English proficiency and speak Spanish, and the percent of households that are linguistically isolated and speak Spanish. Scores describing all Texas census tracts were grouped into statewide quintiles, from one (least ethnically distinct) to five (most ethnically distinct). We define enclaves as all tracts in the highest quintile. The area deprivation index (ADI) is a composite estimator of neighborhood deprivation, based on seventeen items from the U.S. decennial Census or American Community Survey addressing income, education, employment, and housing quality [25], and has been validated at the census tract and block group levels [37].

For children diagnosed in 1995–2004, we used 2000 U.S. Census data to calculate Hispanic enclave and ADI scores; for children diagnosed in 2005 or later, we used 2008–2012 American Community Survey data to compute Hispanic enclave scores, and five-year estimates from the 2007–2011 American Community Surveys to compute ADI. All ADI scores were calculated using the ‘get_adi’ function in the R package ‘sociome’ [38], with Texas as the reference area.

Lastly, using United States Department of Agriculture Rural-Urban Commuting Area (RUCA) codes, we additionally classified each child as residing in a metropolitan (codes 1–3) or non-metropolitan (codes 4–10) tract [39]. For children diagnosed in 2004 or earlier, we used 2000 RUCA codes, whereas for children diagnosed in 2005 or later, we used 2010 RUCA data.

### 2.3. Statistical Analysis

We summarized the distributions of continuous variables by Hispanic enclave score using means and standard deviations (SDs), and differences in the proportions of categorical variables using counts and percentages. We visualized the distribution of enclave index scores across Texas and major metropolitan areas (Austin, Dallas-Fort Worth, Houston, and San Antonio) using the ‘ggmap’ R package [40].

Survival analyses were performed using the R package ‘survival’ [41,42]. We performed Kaplan–Meier analyses to determine whether overall survival (OS) differed between children living vs. not living in enclaves. We estimated 5 year OS and evaluated differences using both the log-rank (sensitive to late differences) and Peto and Peto-modified Gehan-Wilcoxon test (sensitive to early differences) [43]. We did not evaluate cause-specific mortality as nearly all deaths observed (>95%) were attributable to leukemia. We estimated the hazard ratio (HR) and 95% confidence interval (CI) of death for children living (relative to not living) in enclaves using Cox regression, after assessing whether the proportional hazards assumption was upheld using score tests [44] and visual inspection of Schoenfeld residuals. We computed crude models, multivariable models adjusted for sex, age at diagnosis, year of diagnosis, ADI score, metropolitan vs. non-metropolitan residence, and multivariable models additionally adjusted for race/ethnicity. In all models, observations were clustered by tract.

In both Kaplan–Meier and Cox analyses, we first performed comparisons among all children, and, secondly, among Hispanic children specifically. In the former analysis, we aimed to estimate the effect of enclave residence per se on OS; in the latter, we aimed to eliminate or reduce potential residual confounding attributable to differences in clinical and cytogenetic characteristics between Hispanic and non-Hispanic individuals, which may affect prognosis. Additionally, in order to assess whether the association between enclave residence and OS varied by time, we performed stratified analyses of children diagnosed between 1995–2004 and 2005–2011.

All statistical analyses were performed in R v3.6.3 (R Foundation, Vienna, Austria).

## 3. Results

We identified 4083 children diagnosed with ALL and living in Texas, with data on residential address at the time of diagnosis. Among these, *N* = 1223 (30.0%) were residing in a Hispanic enclave at the time of their diagnosis. Hispanics (*N* = 2159) accounted for 52.9% of the study sample, and 50.7% of these individuals (*N* = 1095) were residing in enclaves at diagnosis. Mean ADI score, proportion living in metropolitan areas, and proportion deceased at the end of follow-up were greater among individuals living in enclaves (Table 1). The distributions of sex and mean age at diagnosis were similar and consistent with known incidence patterns for ALL [45].

**Figure 1:**
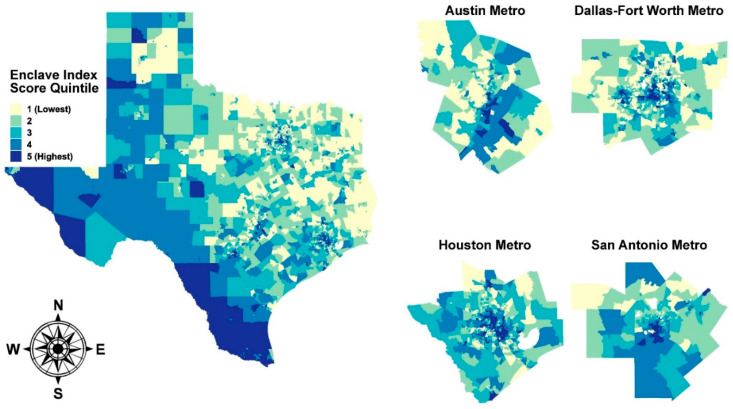
Distribution of Hispanic enclave index scores in Texas and selected metropolitan statistical areas. Enclave index scores were divided into quintiles based on their statewide distribution. For visualization purposes, scores were computed using 2010 U.S. Census data.

Figure 1 shows the distribution of Hispanic enclave index scores across Texas, as well as the Austin, Dallas-Fort Worth, Houston, and San Antonio metropolitan areas. Geographic patterns were evident; enclaves were clustered in regions of south and west Texas proximal the U.S.-Mexico border, as well as in large urban cores.

**Figure 2:**
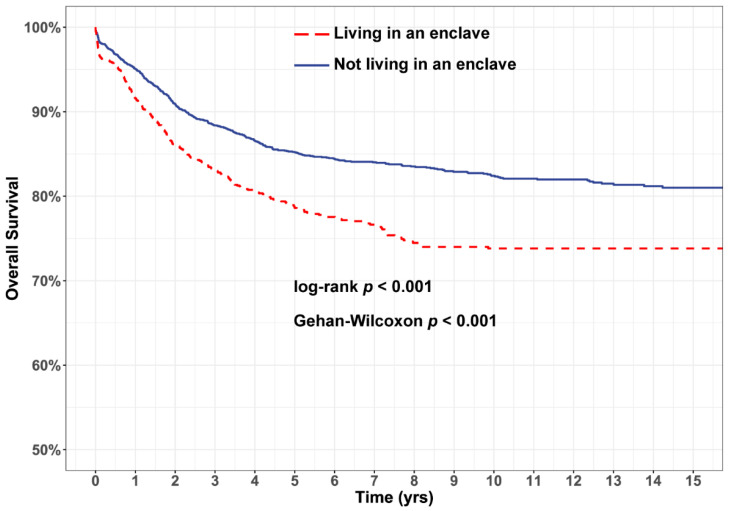
Kaplan–Meier analysis indicates children living in Hispanic enclaves have poorer overall survival. Significant survival disparities were observed for children in Hispanic enclaves at the time of their diagnosis (red), relative to children not in enclaves (blue).

**Figure 3:**
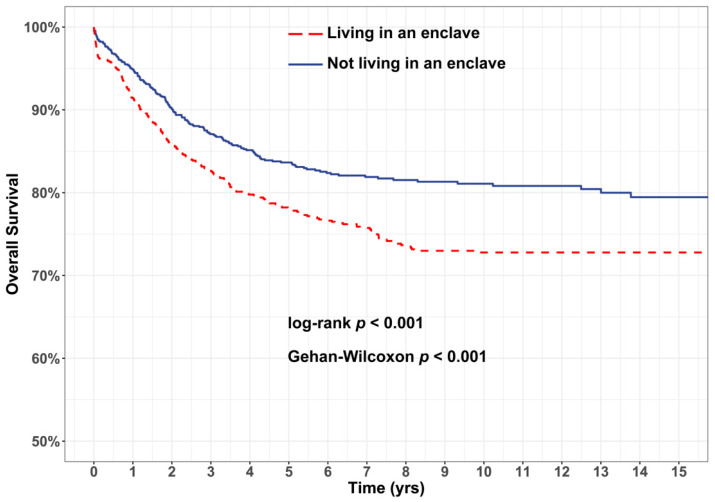
Kaplan–Meier analysis indicates Hispanic children living in enclaves have poorer overall survival than Hispanic children not living in enclaves. Significant survival disparities were observed for Hispanic children living in enclaves at the time of their diagnosis (red), relative to Hispanic children not living in enclaves (blue).

Median (interquartile range) follow-up time from diagnosis among the cohort was 6.7 (8.8) years, and 2479 children (60.7%) were followed for at least 5 years from diagnosis. Kaplan–Meier analysis revealed poorer OS for children living in enclaves (Figure 2, Table 2), with both log-rank and Gehan–Wilcoxon tests indicating statistically significant differences (*p* < 0.001). The estimated 5 year OS was 85.8% (95% CI 84.4–87.3%) among children not living in enclaves and 78.6% (95% CI 76.2–81.0%) among children living in enclaves. Survival disparities persisted in analyses of Hispanic children (Figure 3, Table 2): five-year OS was 84.7% (95% CI 82.1–87.5%) for Hispanic children not living in enclaves, relative to 77.8% (95% CI 75.3–80.5%) for those living in enclaves.

We found no evidence that the proportional hazards assumption was violated. In crude Cox models, residence in an enclave was associated with an increased risk of death, overall (HR 1.52, 95% CI 1.31–1.76) and among Hispanics specifically (HR 1.43, 95% CI 1.19–1.73) (Table 2). The association remained significant in multivariable models adjusted for sex, age at diagnosis, year of diagnosis, neighborhood ADI score, and metropolitan vs. non-metropolitan residence (HR 1.22, 95% CI 1.01–1.49). In the model including all children, we observed a similar association after additional adjustment for the child’s race/ethnicity (HR 1.19, 95% CI 0.97–1.45).

We performed several additional analyses to further evaluate the influence of neighborhood factors on OS for Hispanic children with ALL. First, in order to determine whether results may be driven by the inclusion of children from geographically remote enclaves near the U.S.–Mexico border, we restricted our analyses to children living in metropolitan areas. Risk of death for children living in enclaves remained elevated, although the 95% confidence interval included the null (HR 1.19, 95% CI 0.93–1.53). We estimated the HR of death for Hispanic relative to non-Hispanic children, with and without adjustment for Hispanic enclave index and ADI scores, in order to determine whether the effect of ethnicity was reduced by controlling for neighborhood factors. In the crude Cox model, we observed a 42% (95% CI 1.23–1.65) increased risk of death for Hispanic children compared to White children. This effect was attenuated after adjustment for enclave residence and ADI scores (HR 1.19, 95% CI 1.01–1.41), suggesting that neighborhood factors may contribute to survival disparities for Hispanic children. Interestingly, the hazard of death for Hispanics residing in less ethnically distinct neighborhoods (quintiles 1–3) was similar to that of non-Hispanic Whites in these neighborhoods (HR 1.11, 95% CI 0.86–1.45).

Finally, we performed analyses stratified by year of diagnosis (1995–2004, 2005–2011) (Figure S1, Table S1). During the earlier period, 5 year OS was 9% lower for children living in enclaves relative to children not living in enclaves, and 7.5% lower among Hispanic children specifically (all *p* < 0.001 by log-rank and Gehan–Wilcoxon tests). During the later period, we observed a 2.7% decrement in 5 year OS for children living in enclaves relative to children not living in enclaves, and a 2.8% decrement among Hispanic children specifically (all *p* > 0.05). Likewise, maximally adjusted Cox models indicated an increased risk of death for children living in enclaves (39% in the analysis of all children and 48% in the analysis of Hispanics specifically) during the early period, whereas there were no significant differences during the later period.

## 4. Discussion

We sought to determine whether residence in a Hispanic enclave was associated with overall survival among children with acute lymphoblastic leukemia. Evidence suggests that neighborhood socioeconomic deprivation is associated with inferior survival among children with leukemia [20,21,22,23,46,47], but no studies have investigated the effects of residence in ethnic enclaves in this population. We report that living in an enclave was associated with a 6–7% reduction in the estimated 5 year OS in Kaplan–Meier analyses, and a 20–25% increase in the risk of death in multivariable Cox analyses. Of note, these findings were essentially unchanged in subgroup analyses of Hispanic children. Our findings provide additional evidence that social determinants of health, including neighborhood ethnic and socioeconomic context, are implicated in disparities for children with ALL.

In the present study, the estimated 5 year OS for children living in enclaves was approximately 77–79%. While these rates are excellent relative to many adult malignancies, it is worth noting that 5 year OS among children enrolled on some contemporaneous clinical trials reached 90% [48,49,50]. We estimated 5 year OS among non-Hispanic White children at 86.1% (95% CI 84.8–88.0%), which is similar to other reports [22,51], and scarcely superior to the 83.7% (95% CI 81.4–86.0%) observed among Hispanic children who did not reside in enclaves. Cox analyses provide further support for neighborhood disparities. The risk of death for Hispanic children in the most ethnically distinct neighborhoods (quintile five) was increased by 30%, relative to other Hispanic children. To our knowledge, there are no previous studies against which our findings may be directly compared; however, Abrahão et al. reported a 39% increased hazard of death among Hispanic children with ALL in California between 1988 and 2011 [22]. Using Surveillance, Epidemiology, and End Results (SEER) data, Kadan-Lottick et al. reported a 1.8-fold increased hazard of death (95% CI 1.4–2.4) among Hispanic children diagnosed in 1990–1999 [52]. We observed a 42% increased risk of death for Hispanic children, but this was attenuated after adjustment for enclave and ADI scores, suggesting that neighborhood factors may contribute to ethnic disparities. Furthermore, the risk of death for Hispanics and non-Hispanic Whites residing in less ethnically distinct neighborhoods was similar. Thus, we observed a more pronounced disparity comparing Hispanic children by neighborhood context than comparing non-Hispanic White to Hispanic children within less ethnically distinct neighborhoods. The importance of neighborhood context is further underscored by the fact that, in the present study, >50% of Hispanic children lived in enclaves.

The present study included data from children diagnosed with ALL between 1 January 1995 and 31 December 2011. Nationally, survival for children with ALL improved during this period [53], but the published literature suggests that ethnic disparities persisted. Using SEER data, Kahn et al. reported 5 year OS of 93.3% for non-Hispanic White children and 84.5% for Hispanic children during the years 2000–2003 [54], and Delavar et al. reported an increased risk of death (adjusted hazard ratio (aHR) 1.63, 95% CI 1.50–1.78) for Hispanic children relative to non-Hispanic White children diagnosed with ALL and other cancers highly amenable to treatment from 2000–2016 [55]. In the present study, we observed similar survival for children living in enclaves vs. not living in enclaves for the later period, 2005–2011. Our results may suggest that ethnic disparities in Texas have narrowed over time as survival has improved, but, because not all children diagnosed during the latter half of the study had at least 5 years of follow-up, our results should be interpreted with caution. Notably, this analysis is based on a smaller sample, and data may not capture late mortality attributable to ALL relapse, which is more common among Hispanic children compared to their non-Hispanic White peers [56]. Furthermore, ALL relapse is associated with factors we hypothesize are influenced by neighborhood context (e.g., treatment adherence) [16]. Therefore, stratified analyses may not reflect the full magnitude of disparities for children living in enclaves. As ALL therapy becomes increasingly individualized, there is an opportunity to address disparities through targeted therapies; on the other hand, inequalities in access to care, treatment adherence, and treatment-related toxicities may potentially worsen disparities. Hence, understanding temporal trends in racial/ethnic and socioeconomic disparities for children with ALL is of great importance.

The mechanisms by which ethnic enclaves are associated with or may influence residents’ health are manifold. It is hypothesized that social ties within enclaves offset some of the deleterious effects of economic deprivation and cultural isolation that are common within enclaves, thus leading to better-than-expected health outcomes for their residents. However, in the present study, we observed inferior OS among children in enclaves. Among adults, residence in an enclave has been associated with more advanced cancer stage at presentation [31,32,57], which would be anticipated to lead to inferior outcomes. It is unclear whether this phenomenon would apply to ALL, a rapidly progressive cancer, which is invariably fatal if untreated. Hispanic enclaves are often characterized by socioeconomic deprivation; in the present study, this was demonstrated by their greater mean ADI scores. Socioeconomic status, at the neighborhood and individual levels, is associated with outcomes for children with ALL, a disease with complex, protracted therapy [20,21,22,23]. The financial toxicity associated with an ALL diagnosis is considerable, and substantial proportions of families report changes in employment or housing [14,15]. Such hardship may disproportionately affect residents of enclaves, who may have limited housing, employment, and transportation options, negatively impacting their access and/or adherence to care. In support of this assertion, we note that differential adherence to oral maintenance chemotherapy has been observed in racial/ethnic minority populations and among children with lower parental educational attainment [16,17]. While >90% of children in enclaves also lived in urban areas, we did observe that by removing the limited number of children residing within rural areas from the analysis somewhat reduced the magnitude of the association between enclave residence and OS. As rural areas tend to be geographically remote, this finding may implicate differential access to care as one mechanism by which enclaves are associated with OS. Finally, language isolation, financial hardship, and other experiences associated with living in an enclave, are significant psychosocial stressors and may contribute to poor health outcomes in and of themselves.

Our study has several important strengths. We analyzed data on >4000 ALL cases in TCR, a population-based registry which is gold-certified by the North American Association of Central Cancer Registries (NAACR) for data quality, completeness, and timeliness. Texas is an excellent setting for studies about residents of ethnic enclaves; the state has a large Hispanic population, distributed across densely populated urban and expansive rural areas. We used a multidimensional measure to define ethnic enclaves, based on the most widely used measure of ethnic enclaves in the cancer literature with minor modifications [35,36], and adjusted for neighborhood socioeconomic status (ADI) and child race/ethnicity. Our large sample enabled us to perform stratified analyses among Hispanic children, in which the potential contributions of ethnic differences in disease characteristics to outcomes were reduced.

With respect to limitations, we were unable to adjust for certain individual and sociodemographic characteristics, such as insurance status, parental education and employment, and ALL cytogenetics, as these data were not collected. While our results should be interpreted in light of this potential residual confounding, we note that studies of ALL outcomes show similar associations for individual- and area-level SES and education measures. Similarly, we were unable to assess whether Hispanic children living in enclaves differed from those in less ethnically distinct neighborhoods with respect to these and other characteristics (e.g., nativity, national origin, and degree of Amerindian admixture). However, since we performed our study in a large, unselected population, we anticipate that our findings are broadly applicable to Hispanic populations in the southwestern United States and to children with ALL. Due to the nature of the data, we were unable to assess specific causes of mortality (e.g., ALL relapse and treatment-related toxicity). Finally, as they were collected from 1995–2012, our data may not reflect current neighborhood disparities or survival rates among children with ALL.

## 5. Conclusions

Our findings suggest that children living in Hispanic enclaves experience poorer overall survival after a diagnosis of ALL. With respect to ALL, Hispanic children bear the dual burden of higher incidence and poorer survival, but relatively little research has been performed to characterize and address the social determinants of these disparities. Future investigations should evaluate potential mechanisms or explanations underlying this association, which may relate to financial and material hardship, psychosocial stressors, access to healthcare, or cultural and language isolation.

## Figures and Tables

**Table 1:** Demographic characteristics of children with acute lymphoblastic leukemia (ALL) according to Hispanic enclave scores.

Characteristic	Quintile 1		Quintile 2		Quintile 3		Quintile 4		Quintile 5	
	*N*	%	*N*	%	*N*	%	*N*	%	*N*	%
Cases	611	-	699	-	734	-	816	-	1223	-
Deceased	84	13.7	119	17.0	108	14.7	144	17.6	280	22.9
Sex										
Male	350	57.3	414	59.2	391	53.3	447	54.8	699	57.2
Female	261	42.7	285	40.8	343	46.7	369	45.2	524	42.8
Race/ethnicity										
Non-Hispanic White	448	73.3	448	64.1	338	46.0	194	23.8	90	7.4
Non-Hispanic Black	43	7.0	44	6.3	75	10.2	70	8.6	26	2.1
Hispanic	79	12.9	172	24.6	290	39.5	523	64.1	1095	89.5
Non-Hispanic Other	41	6.7	35	5.0	31	4.2	29	3.6	12	1.0
Residing in a metro area ^1^	530	86.7	592	84.7	602	82.0	686	84.1	1129	92.3
**Characteristic**	**Mean**	**SD**	**Mean**	**SD**	**Mean**	**SD**	**Mean**	**SD**	**Mean**	**SD**
Area deprivation score	79.9	17.5	85.3	14.1	94.4	12.6	104.7	12.0	121.9	12.8
Age at Diagnosis (yrs)	6.3	5.0	6.6	5.3	6.8	5.1	6.9	5.3	6.9	5.2

^1^ For children diagnosed in 1999–2004, residence in a 2000 U.S. census tract with a rural–urban commuting area (RUCA) code 1–3. For children diagnosed in 2005–2011, residence in a 2010 U.S. census tract with RUCA code 1–3. SD, standard deviation.

**Table 2:** Estimated 5-year overall survival and hazard of death for children living in Hispanic enclaves.

Group	*N* at Risk (5 year OS)	5 Year OS % (95% CI)	cHR (95% CI)	aHR (95% CI) ^1^	aHR (95% CI) ^2^
All children					
Not living in an enclave	1790	85.8 (84.4–87.3)	1.00	1.00	1.00
Living in an enclave	691	78.6 (76.2–81.0)	1.52 (1.31–1.76)	1.22 (1.01–1.49)	1.19 (0.97–1.45)
Hispanic children					
Not living in an enclave	626	83.7 (81.4–86.0)	1.00	1.00	-
Living in an enclave	608	77.8 (75.3–80.5)	1.43 (1.19–1.73)	1.30 (1.03–1.65)	-

^1^ Adjusted for sex, age at diagnosis, year of diagnosis, metropolitan vs. non-metropolitan residence, and census tract area deprivation index score, with observations clustered by census tract. ^2^ Adjusted for race/ethnicity, sex, age at diagnosis, year of diagnosis, metropolitan vs. non-metropolitan residence, and census tract area deprivation index score, with observations clustered by census tract. OS, overall survival; CI, confidence interval; cHR, crude hazard ratio; aHR, adjusted hazard ratio.

## Data Availability

Restrictions apply to the availability of these data. Data were obtained from the Texas Department of State Health Services, and, to protect privacy and confidentiality, cannot be made available to third parties. Investigators may apply to the Texas Cancer Registry to access the underlying data.

## Supplementary Materials

The following are available online at https://www.mdpi.com/article/10.3390/ijerph18179273/s1, Figure S1: Kaplan–Meier analysis indicates children living in Hispanic enclaves experienced poorer overall survival during the earlier but not the latter half of the study period. Table S1: Estimated 5 year overall survival and hazard of death for children living in Hispanic enclaves, stratified by year of diagnosis.
