# Supplementary material for: Residence in a Hispanic Enclave Is Associated with Inferior Overall Survival among Children with Acute Lymphoblastic Leukemia
Source: Int J Environ Res Public Health. 2021 Sep 2;18(17):9273. doi: 10.3390/ijerph18179273 (PMC8430860; doi:10.3390/ijerph18179273)
Supplement: Supplementary file 1 [file ijerph-18-09273-s001.zip › ijerph-1338475-supplementary.pdf]

International Journal of
Environmental Research and Public Health

MDPI

Supplementary Material

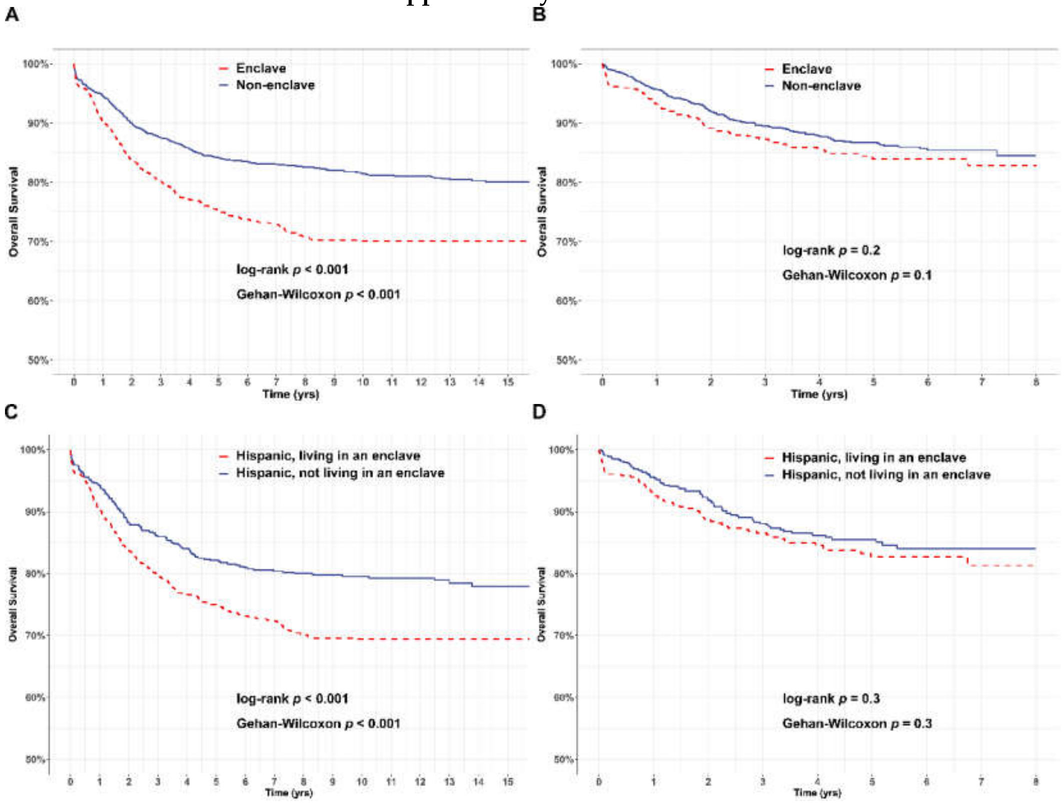

Figure S1. Kaplan-Meier analysis indicates children living in Hispanic enclaves experienced poorer overall survival during the earlier but not the latter half of the study period. Survival estimates for children living in enclaves are shown in red; survival estimates for children not living in enclaves are shown in blue. Comparison of five-year overall survival for (**A**) children living in enclaves vs. not, 1995–2004. (**B**) Hispanic children living in enclaves vs. not, 1995–2004. (**C**) children living in enclaves vs. not, 2005–2011. (**D**) Hispanic children living in enclaves vs. not, 2005–2011.

Table S1. Estimated 5-year overall survival and hazard of death for children living in Hispanic enclaves, stratified by year of diagnosis.

Study sample and diagnosis period	*N* at risk (5-year OS)	5-year OS % (95% CI)	cHR (95% CI)	aHR (95% CI) [1]	aHR (95% CI) [2]
Children Diagnosed 1995–2004					
All children					
Not living in an enclave	1,300	84.1 (82.3–86.0)	1.00	1.00	1.00
Living in an enclave	513	75.1 (71.9–78.4)	1.67 (1.39–1.99)	1.42 (1.11–1.82)	1.39 (1.07–1.79)
Hispanic children					
Not living in an enclave	427	82.1 (78.9–85.5)	1.00	1.00	-
Living in an enclave	459	74.6 (71.2–78.2)	1.53 (1.21–1.93)	1.48 (1.10–2.00)	-
Children Diagnosed 2005–2011					
All children					
Not living in an enclave	484	86.7 (84.7–88.8)	1.00	1.00	1.00
Living in an enclave	178	84.0 (80.6–87.5)	1.21 (0.91–1.61)	0.92 (0.64–1.31)	0.89 (0.62–1.27)
Hispanic children					
Not living in an enclave	199	85.5 (82.3–88.8)	1.00	1.00	-
Living in an enclave	149	82.7 (79.0–86.7)	1.19 (0.85–1.65)	1.07 (0.71–1.61)	-

[1] Adjusted for sex, age at diagnosis, year of diagnosis, metropolitan vs. non-metropolitan residence, and census tract Area Deprivation Index score, with observations clustered by census tract. [2] Adjusted for race/ethnicity, sex, age at diagnosis, year of diagnosis, metropolitan vs. non-metropolitan residence, and census tract Area Deprivation Index score, with observations clustered by census tract.
